# Pharmacological effects of specialized pro-resolving mediators in sepsis-induced organ dysfunction: a narrative review

**DOI:** 10.3389/fimmu.2024.1444740

**Published:** 2024-09-20

**Authors:** Shujun Sun, Dong Yang, Jing Lv, Haifa Xia, Zhangyan Mao, Xiangdong Chen, Yafen Gao

**Affiliations:** ^1^ Department of Anesthesiology, Union Hospital, Tongji Medical College, Huazhong University of Science and Technology, Wuhan, China; ^2^ Institute of Anesthesia and Critical Care Medicine, Union Hospital, Tongji Medical College, Huazhong University of Science and Technology, Wuhan, China; ^3^ Key Laboratory of Anesthesiology and Resuscitation (Huazhong University of Science and Technology), Ministry of Education, Wuhan, China; ^4^ Department of Pain, Union Hospital, Tongji Medical College, Huazhong University of Science and Technology, Wuhan, China

**Keywords:** specialized pro-resolving mediators (SPMs), sepsis, lipoxin, resolvin, protectin, maresins

## Abstract

Sepsis is a life-threatening syndrome of organ dysfunction, characterized by uncontrolled inflammatory response and immune dysregulation, often leading to multiple organ failure and even death. Specialized pro-resolving mediators (SPMs), which are typically thought to be formed via consecutive steps of oxidation of polyenoic fatty acids, have been shown to suppress inflammation and promote timely resolution of inflammation. They are mainly divided into four categories: lipoxins, resolvins, protectins, and maresins. The SPMs may improve the prognosis of sepsis by modulating the immune and inflammatory balance, thereby holding promise for clinical applications. However, their biosynthetic and pharmacological properties are very complex. Through a literature review, we aim to comprehensively elucidate the protective mechanisms of different SPMs in sepsis and its organ damage, in order to provide sufficient theoretical basis for the future clinical translation of SPMs.

## 1 Introduction

Sepsis is a life-threatening organ dysfunction syndrome resulting from a dysregulated host response to infection, and is a major public health problem affecting the general level of human health (1). Epidemiological surveys have shown that there are nearly 50 million cases of sepsis globally each year, of which approximately 11 million die, with the attendant healthcare costs of tens of billions of dollars (2). An important pathophysiological process in sepsis is uncontrolled inflammatory immunity, which is frequently accompanied by a confluence of over-activation of inflammatory hyperactivation and immunosuppression. In this scenario, pathogens evade the host’s defense mechanisms to multiply continuously, stimulating and damaging host cells ultimately leading to an inability to regain homeostasis, which then results multi-organ dysfunction (3, 4). Despite the great progress in anti-infective, fluid resuscitation and organ function support techniques, they do not intervene at the source of immune disorders. It is urgent to explore ways to regulate inflammatory immune disorders to improve the prognosis of patients in sepsis.

Specialized pro-resolving mediators (SPMs) are a class of lipid mediators derived from polyunsaturated fatty acids with anti-inflammatory and pro-resolving effects (5). In 1984, Serhan et al. (6) identified a new class of substances called lipoxins (LX) in the metabolism of arachidonic acid (AA). Besides AA derivatives, resolvins, protectins and maresins are found among the metabolites of n-3 polyunsaturated fatty acids (PUFAs). Resolvins are classified into E-series (RvE) and D-series (RvD) based on the usage of (eicosapentaenoic acid) EPA and (docosahexaenoic acid) DHA as substrate, respectively. Maresin (MaR), first identified in macrophages, mainly includes MaR1 and MaR2. In-depth reviews have been published on the biosynthesis, structure, and function of SPMs (7, 8). Noteworthy is the considerable debate on the formation of specific SPMs in the human body as well as in general. There seems to be a recent consensus that there is no significant formation of trihydroxylated SPMs in biological samples (9, 10). Several receptors are currently reported to bind SPMs, such as LXA4/formyl peptide receptor 2 (ALX/FPR2), RvD1/G protein-coupled receptor 32 (GPR32), RvD2/GPR18, RvE1/Chemerin Receptor 1 (ChemR23), and MaR1/leucine-rich repeat-containing G protein-coupled receptor 6 (LGR6) (5, 11). However, the functional validation of these receptors is still a subject of debate. Despite initial reports, not all SPM/receptor bindings have been consistently reproduced, and also functional responses such as those mediated by FPR2 or ChemR23 to their respective SPMs could not be reproduced (9, 12, 13). The field is evolving, and further research is needed to fully understand the role of these receptors in mediating the effects of SPMs.

A large number of studies have demonstrated the protective effects of SPMs against different sepsis-induced organ dysfunction (14–16). In our review, the protective effects of SPMs in sepsis-induced organ dysfunction are summarized (**Tables 1**–**5**), aiming to provide new ideas for the treatment of sepsis, as well as more evidence for the development and clinical application of SPMs.

**Table 1:** Protective mechanisms of SPMs against septic lung injury.

SPMs	Model	concentration/dose and application	Mechanisms of intervention	Effect/outcome	Reference
LXA4	CLP	LXA4(0.1 mg/kg)	Pulmonary edema	AFC↑, W/D ratio of lung tissues↓	(25)
			Inflammatory cells/cytokines	Total cell count in BALF and PMN percentage↓, TNF-α↓	
			MAPK/oxidative stress	P-p38↓, MPO↓	
AT-LXA4	LPS Intratracheal Injection	AT-LXA4 (100 μg/kg) injected via the external jugular vein	ECM remodelling and tissue stiffness	ECM proteins fibronectin, collagen I, ECM crosslinker enzyme and lysyl oxidase↓	(27)
			Adhesion molecule/cytokines	VCAM1 and ICAM1↓, IL-8↓	
	LPS Intratracheal Injection	100 to 5000 ng/mouse of AT-LXA4	Neutrophil-platelet	Platelet activation and NPA↓	(26)
RvD1	CLP	RvD1 (10 ng/g body weight) by penile vein injection	Inflammation response	STAT3/NF-κB/ERK/p38↓, SIRT1↑, TNF-α, IL-6 and IL-1β↓	(28)
			Pulmonary exudate/oxidative stress	Albumin↓, MPO↓	
			Leukocyte	Adhesion and proportion↓	(29)
			Adhesion molecule/oxidative stress	ICAM-1↓, MPO↓	
RvD2	Secondary lung infection after CLP	48 h after CLP, RvD2 (100 ng/mouse) via the tail vein	Bacterial clearance	Splenic neutrophils and MDSCs↑	(30)
			Pulmonary infection/cytokine	Pulmonary bacterial load↓, non-inflammatory macrophages↑, IL-23↓	
PDX	CLP	low dose (500 ng) or high dose (1000 ng) of PDX injected intraperitoneally	Pulmonary edema/exudate	W/D ratio of lung tissues and protein↓	(31)
			Inflammation response	PPARγ↑, NF-κB↓, TNF-α, IL-6, MCP-1 and IL-1β↓, IL-10↑	
			Inflammatory cells	Leukocyte recruitment and neutrophil infiltration↓	
PCTR1	LPS intraperitoneal injection	PCTR1 100 ng/mouse administrated intraperitoneally	Pulmonary edema	W/D ratio of lung tissues and EBD↓	(32)
			Inflammation response	SIRT1↑, NF-κB↓, TNF-α, IL-6 and IL-1β↓	
			Endothelial cells	HS and SDC-1 in lungs↑, HPA↓, EXT-1↑	
		PCTR1(200ng/mouse) injected intravenously	Ferroptosis/mitochondria	Fe2+, PTGS2 and ROS↓, GSH and GPX4↑	(33)
			Signaling pathway	ALX, PKA and CREB↑	
MaR1	CLP	MaR1 1 ng injected via the tail vein	Inflammatory response	Neutrophils↓, NF-κB↓, TNF-α, IL-6 and IL-1β↓	(34)
			Bacterial clearance	Bacterial load and LPS↓	
		MaR1 (100 ng/ mouse) injected into peritoneum	Mitochondria	NOX↓, CAT and SOD↑, ROS and mtO2↓ ALX, cAMP and mtDNA↑	(35)
MCTR1	LPS intraperitoneal injection	MCTR1 (100 ng/ mouse)	Inflammatory response	TNF-α, IL-6 and IL-1β↓	(36)
			Endothelial cells	HS, syndecan-1 and HPA↓	
			Signaling pathway	SIRT1↑, NF-κB↓	

bronchoalveolar lavage fluid (BALF); Alveolar fluid clearance (AFC); extracellular matrix (ECM); Vascular Cell Adhesion Molecule 1 (VCAM1); Intercellular Adhesion Molecule 1 ( ICAM1); neutrophil-platelet aggregates (NPA); wet/dry (W/D); evans blue dye (EBD); heparin sulphate (HS); hyaluronic acid (HA); and syndecan-1 (SDC-1); heparanase (HPA); exostosin-1 (EXT-1); cAMP-response element binding protein (CREB); prostaglandin-endoperoxide synthase 2 (PTGS2); glutathione (GSH); Glutathione peroxidase 4 (GPX4); NADPH oxidase (NOX); catalases (CAT); superoxide dismutase (SOD). ↑ represents an increase in level; ↓ represents a decrease in level.

**Table 2:** Protective mechanisms of SPMs against septic kidney injury.

SPMs	Model	concentration/dose and application	Mechanisms of intervention	Effect/outcome	Reference
LXA4	CLP	100 µg/kg LXA4 pre-injected intravenously	NF-κB	Phosphorylation p65↓, IL-6, TNF-a and HMGB1↓	(42)
			P53/p21 senescence pathway	Tubular Epithelial Cell Senescence↓, p53 and p21↓	
RVD1	LPS intraperitoneal injection	RvD1 5 μg/kg injected intraperitoneally	NF-κB	Phosphorylation p65↓	(40)
			Apoptosis	Caspase-3 activity↓, renal cell apoptosis↓	
AT-RVD1	LPS intraperitoneal injection	AT-RvD1 (1μg/mouse ) intraperitoneally	Inflammatory response	Neutrophil infiltration↓, ICAM-1 and VCAM-1↓, IL-6↓, p65, STAT3 and ERK phosphorylation↓	(38)
			Intercellular junction	Claudin-4↑, disruption of barrier function↓	
	Intraperitoneal injection of BSA 15 days after CLP (subclinical acute kidney injury animal model)	5 μg/kg ATRvD1, i.v	Inflammatory response	Iba1+ cells↓, IL-1β, TNF-α, IL-4 and IL-10↓	(43)
			Fibrotic progression	Collagen deposition and TGFβ↓, MMP-3 and MMP-9↓	
			Apoptosis	Cleaved caspase-3 in renal tissue↓	
MaR1	CLP	MaR1 1 ng injected via the tail vein (34) a low dose (0.5 ng) or high dose (1 ng) of MaR1 injected via the tail vein (39)	Cytokines	IL-1β, TNF-α and IL-6↓, IL-10↑	(39)
			NF-κB/MAPK/STAT3	Phosphorylation of p65, JNK, ERK, p38, and STAT3↓	
			Bacterial burden	Blood and abdominal bacterial load↓	
	LPS intraperitoneal injection	MaR1 (5 μg/kg) intraperitoneally	NOX4/ROS/NF-κB pathway	NOX4↓, ROS↓, activation of the NF-κB p65↓	(41)
MCTR1	CLP	MCTR1 (200 ng/mouse, iv), qd or bid	Antioxidant stress	Expression of Nrf2↑	(14)
			Ferroptosis	GPX4↑, PTGS2↓, MDA, non-heme iron and heme-iron↓, GSH ↑	

bovine serum albumin (BSA); ionized calcium-binding adoptermolecule 1 (Iba1+); glutathione peroxidase 4 (GPX4); prostaglandin-endoperoxide synthase 2 (PTGS2); The once-daily administration mode (qd); The twice-daily administration mode (bid); mitogen-Activated Protein Kinase (MAPK); nuclear Factor-κB (NF-κB); ↑ represents an increase in level; ↓ represents a decrease in level.

**Table 3:** Protective mechanisms of SPMs against septic liver injury.

SPMs	Model	concentration/dose and application	Mechanisms of intervention	Effect/outcome	Reference
LXA4	LPS intraperitoneal injection	LXA4 10μg/kg injected intraperitoneally	TLR4 signaling pathway	IL-6 and TNF-α↓, expression of TLR4 and TRAF6↓	(45)
RVD1	LPS/d-GalN mouse model	RvD1 (0.1 or 1 μg) injected intraperitoneally	Inflammatory cells/cytokines	Neutrophil population↓, HMGB1, TNF-α, IL-6, IL-10 and MCP-1↓	(44)
			Apoptosis	Tunel hepatocytes↓	
MaR1	CLP	1 ng of MaR1 injected via the tail vein (34) 100 ng/mouse of MaR1 intraperitoneally (48)	Inflammatory response	NF-κB activation↓, IL-1β, TNF-α and IL-6↓	(34) (48)
	LPS/D-GalN mouse model	MaR1 (100ng, i.v.)	Lipid Peroxidation	GSH and GSH/GSSG↑, ROS↓	(47)
			Ferroptosis	Nrf2, HO-1 and GPX4↑, ferroptosis-induced liver injury↓	
		MaR1 (50 and 100 ng) by tail vein injection	Inflammatory response	MAPK/NF-κB activation↓, IL-1β, TNF-α and IL-6↓	(46)
			Pyroptosis	NLRP3 and p30-GSDMD↓	

D-galactosamine (D-GalN); TNF receptor associated factor 6 (TRAF6); high mobility group protein B1 (HMGB1); macrophage chemotactic protein (MCP)-1; glutathione/oxidized glutathione (GSH/GSSG); NLR family pyrin domain-containing 3 (NLRP3); Gasdermin D (GSDMD); mitogen-Activated Protein Kinase (MAPK); nuclear Factor-κB (NF-κB); ↑ represents an increase in level; ↓ represents a decrease in level.

**Table 4:** Protective mechanisms of SPMs against septic heart injury.

SPMs	Model	concentration/dose and application	Mechanisms of intervention	Effect/outcome	Reference
R_V_D1	LPS intraperitoneal injection	RvD1 (5 ug/kg) injected intraperitoneally	Cardiac function	Left ventricular diameter↓, left ventricular contractility↑, CK-MB↓, LDH↓	(52)
			Myocardial Apoptosis	Bax↓, caspase 3↓, Bcl-2↑	
			Inflammatory response	IL-1β↓, IL-6↓, MCP-1↓, infiltration of neutrophils and M1 macrophages↓	
			MAPK and NF-κB signaling pathways	Phosphorylation of P38, JNK, ERK and p65↓	
R_V_E1	LPS intraperitoneal injection	pretreated i.p. with RvE1 (25 μg/kg)	Inflammatory cells/cytokines	Infiltration of CD68+ macrophages and Ly6G+ neutrophils↓, promote macrophage polarization toward the M2 phenotype, IL-1β, IL-6 and MCP-1↓	(53)
			Cyclooxygenase and lipoxygenase	COX-1 and 5-LOX↑, 15-LOX↓	
			Myocardial Apoptosis	Bax and caspase 3↓, Bcl-2↑	
	CLP	RvE1 (1 μg/mouse i.v.)	Cardiac Akt Phosphorylation	Phosphorylation of Akt↑	(54)
			Bacterial Clearance	Peritoneal bacterial load↓, MHCII- macrophage↑, BMDMs phagocytosis of E.coli ↑	
	LPS intraperitoneal injection/CLP	pretreated i.p. with RvE1 (25 μg/kg) (53) RvE1 (1 μg/mouse i.v.) (54)	Cardiac function	LVEF and LVFS↑, LVEDD, LVESD, CK-MB and LDH↓, fractional area change↑	(53) (54)
			MAPK and NF-κB signaling pathways	Phosphorylation of P38, JNK, ERK and p65↓	
MaR1	LPS intraperitoneal injection	100 ng of MaR1 injected intraperitoneally	Cardiac function	LVEF and LVFS↑, CK-MB and LDH↓	(60)
			Inflammatory cells/response	M1 macrophage↓, M2 macrophage↑, phosphorylation of p65↓, proinflammatory cytokine↓	
			Oxidative stress	Nrf2 and HO-1 activation↑, SOD and GSH↑, MDA↓	
			Apoptosis	C-caspase3 and Bax↓, Bcl-2↑	
MCTR1	LPS intraperitoneal injection	MCTR1 0.15 nmol /mouse or 0.3 nmol /mouse i.v. via caudal vein	Mitochondrial mass/function/biogenesis	COX 1 and VDAC-1↑, content of ATP and complex I-IV activity↑, Sirt1, PGC1α, NRF-1, NRF-2 and TFAM↑	(61)
			Neutrophil Infiltration/chemokines	Ly6G expression and MPO activity↓, CXCL1 and G-CSF↓	
			Inflammatory cells/cytokines	γδ T↓, IL-17A↓	

left ventricular end-diastolic diameter (LVEDD); lV end-systolic diameter (LVESD); lV ejection fraction (LVEF); lV fractional shortening (LVFS); lactate dehydrogenase (LDH); creatine kinase myocardial bound (CK-MB); mitogen-Activated Protein Kinase (MAPK); nuclear Factor-κB (NF-κB); bone marrowderived macrophages (BMDMs); cytochrome c oxidase 1 (COX 1); voltage-dependent anion-selective channel 1 (VDAC-1); peroxisome proliferator-activated receptor gamma coactivator-1 α (PGC1α); chemokine (C-X-C motif) ligand 1 protein (CXCL1); granulocyte colony-stimulating factor (G-CSF); interleukin-17A (IL-17A). ↑ represents an increase in level; ↓ represents a decrease in level.

**Table 5:** Protective mechanisms of SPMs against septic brain injury.

SPMs	Model	concentration/dose and application	Mechanisms of intervention	Effect/outcome	Reference
AT-LXA4	LPS intervention on BV2 cells	incubated with different concentrations (1 nM, 10 nM or 100 nM) of AT-LXA4 for 30 min	Inflammatory cytokines	NO, IL-1β and TNF-α↓	(63)
			NF-κB/MAPK/AP-1 pathway	Activation↓	
R_V_D1	LPS intervention on BV2 cells	incubated with 10 nM of R_V_D1 for 30 min	Pro-inflammatory marker mRNA expression	IL-6, TNF-α, IL-1β and CD86↓	(66)
			Gene expression of miRNAs	MiR-155, miR-21 and miR-146 expression↑, miR-219 expression↓	
	CLP	RvD1 (5 μg/kg) was injected by caudal vein	Brain function	Learning and cognitive ability↑	(65)
			Inflammatory cells/cytokines	Microglia activity↓, IL-6, TNF-α and IL-1β↓	
			NF-κB/MAPK/STAT pathways	Activation↓	
R_V_E1	LPS intervention on BV2 cells	incubated with 10 nM of R_V_E1 for 30 min	Pro-inflammatory marker mRNA expression	IL-6 and IL-1β↓	(66)
			NF-κB pathway	Activation↓	

mitogen-Activated Protein Kinase (MAPK); nuclear Factor-κB (NF-κB); signal transducer and activator of transcription (STAT). ↑ represents an increase in level; ↓ represents a decrease in level.

## 2 Pharmacological effects of SPMs in sepsis-induced acute lung injury

Sepsis frequently impacts the lungs, often resulting in acute lung injury (ALI)/acute respiratory distress syndrome (ARDS), with over 200,000 cases annually in the United States (17). Shi Y et al. (18) reported that 48.6% of septic patients in China developed ALI in the ICU. Despite advancements in ALI/ARDS management, the mortality rate for sepsis-induced cases remains high, underscoring the severity of ALI/ARDS in sepsis (19). Sepsis-induced ALI (SALI) involves complex mechanisms such as imbalanced inflammatory responses, endothelial barrier disruption and alveolar epithelial damage (20–22). Current treatments for SALI include aggressive antibiotics, respiratory and nutritional support and fluid management (23, 24), yet an effective therapy is still lacking. Recent studies focusing on SPMs in preclinical animal models have shown promise (**Table 1**), emphasizing the need for further research into SPMs’ therapeutic mechanisms in SALI.

LXs have demonstrated therapeutic potential in SALI. LXA4 has been shown to enhance alveolar fluid clearance and reduce pulmonary edema, as indicated by a decreased wet/dry weight (W/D) ratio. LXA4 also diminishes bronchoalveolar lavage fluid (BALF) cell counts, particularly polymorphonuclear neutrophils, and reduced levels of TNF-ɑ and IL-6, along with myeloperoxidase (MPO) activity, contributing to improved 7-day survival rates in SALI rats, The p38/MAPK pathway is implicated in LXA4’s protective effects (25). Aspirin-triggered lipoxin A4 (AT-LXA4) mitigated lung injury by inhibiting neutrophil-platelet aggregation, a key factor in SALI’s inflammatory response (26). Furthermore, in a mouse model of LPS-induced lung injury, LPS damaged vascular endothelial cells leading to increased perivascular stiffness and stimulated the expression of extracellular matrix (ECM) proteins fibronectin, collagen I and ECM crosslinker enzyme, lysyl oxidase. In contrast, LXA4 mitigated vascular endothelial damage, reduced the associated inflammatory response, restored the lung compliance (27). Resolvins are lipid mediators derived from EPA and DHA. RvD1 exerted anti-inflammatory effects in a cecal ligation and puncture (CLP) mouse model by reducing levels of TNF-ɑ, IL-1β and IL-6, enhancing silent information regulator 1 (SIRT1) expression and inhibiting the activation of NF-κB, STAT3, ERK and p38, improved survival (28). The combination of RvD1 with Xuebijing diminished MPO activity and intercellular adhesion molecule 1 (ICAM-1) expression, thereby reducing leukocyte adhesion and lung injury in septic mice (29). In a 2-hit model of sepsis with secondary lung infection, RvD2 enhanced myeloid-derived suppressor cells (MDSC) accumulation in the spleen and increased the presence of non-inflammatory alveolar macrophages, facilitating bacterial clearance from both blood and lungs (30). Additionally, RvD2 significantly lowered the concentration of IL-23 in lung lavage fluid, a cytokine critical to the Th-17 inflammatory pathway, contributing to the protective effect against secondary pulmonary infections. Protectins also had a protective effect on SALI (31–33). PDX has been shown to decrease pro-inflammatory cytokines IL-1β, IL-6, TNF-ɑ, and MCP-1, and increase the anti-inflammatory factor IL-10 in BALF. This modulation is associated with PPARγ upregulation and inhibiting NF-κB p65 phosphorylation, leading to reduced lung inflammation and improved lung permeability. Protectin conjugates in tissue regeneration 1 (PCTR1) restored endothelial glycocalyx loss by downregulating heparanase (HPA) and upregulating exostatin-1 (EXT-1), enhancing lung function and the survival rate of SALI mice. Additionally, PCTR1 mitigated mitochondrial damage and reactive oxygen species (ROS) production by decreasing Fe^2+^ and prostaglandin-endoperoxide synthase 2 (PTGS2) levels and by increasing glutathione (GSH) and glutathione peroxidase 4 (GPX4), thereby protecting against LPS-induced ALI through the inhibition of ferroptosis. Maresins is a newly discovered family of lipid mediators synthesized from DHA by macrophages. MaR1 reduced levels of IL-6, TNF-ɑ and IL-1β, decreased bacterial load, and attenuated lung injury in CLP mice (34), partly through the inhibition of NF-κB pathway activation. Additionally, MaR1 mitigated lung injury in CLP mice by attenuating mitochondrial dysfunction through regulating ROS production (35). Maresin conjugates in tissue regeneration 1 (MCTR1) attenuated endothelial glycocalyx injury and improved survival in SALI mice in addition to decreasing serum levels of inflammatory cytokines (TNF-ɑ, IL-1β and IL-6). The mechanism may be related to MCTR1 upregulating the expression SIRT1 and reducing the phosphorylation of NF-κB p65, thereby downregulating HPA (36). The authors suggested that ALX is associated with the protective effect, but its role has not been confirmed using ALX knockout mice (35, 36). SPMs hold significant therapeutic potential for addressing SALI through various key mechanisms. Advancing our knowledge in this area can deepen the understanding of the therapeutic significance of SPMs and expand their potential applications in a range of lung diseases.

## 3 Pharmacological effects of SPMs in sepsis-induced acute kidney injury

Sepsis-induced acute kidney injury (SAKI) is a common complication of sepsis, accounting for 26% to 50% of all AKI, and AKI is an independent risk factor for the increased risk of death in patients with sepsis (37). The pathogenesis of SAKI is complex and involves multiple factors, such as inflammation, renal tubular epithelial cell apoptosis, oxidative stress, and ferroptosis, etc., which can interact with each other and affect the progression of SAKI.

In recent years, research into the role of SPMs in SAKI has focused on their interaction with cytokines, chemokines, and ROS, as well as the exploration of their signaling pathways, including the IL-6, TLR4/NF-κB, and MAPK pathways. In a murine model of LPS-induced acute kidney injury, AT-RvD1 alleviated the inflammatory response and protected kidney function by reducing the expression of ICAM-1 and vascular cell adhesion protein 1 (VCAM-1), inhibiting IL-6 and IL-6-related inflammatory pathways (STAT3 and ERK phosphorylation), and reducing the phosphorylation and activation of NF-κB (38). Similarly, MaR1 also inhibited the NF-κB/STAT3/MAPK signaling pathway to suppress the inflammatory response, thereby dose-dependently mitigating SAKI (39). AT-RvD1 also stabilized the expression level of claudin-4, alleviated the barrier damage in the kidney, and further prevented the infiltration of inflammatory cells (38). RvD1 inhibited the entire pathway from TLR4, MyD88, NF-κB to TNF-α induced by LPS, reduced the inflammatory response and renal cell apoptosis, and thus protected against LPS-induced AKI (40). Mitochondria are the main source and primary target of intracellular ROS. NADPH oxidase 4 (NOX4), a major subtype in the kidney, is highly expressed and increasingly recognized as a major cause of elevated ROS levels, which play a crucial role in mediating mitochondrial dysfunction and cell apoptosis. MaR1 effectively inhibited the NOX4/ROS/NF-κB p65 signaling pathway and reduced kidney inflammation, cell apoptosis, oxidative stress and mitochondrial dysfunction, ultimately protecting against LPS-induced AKI (41).

Oxidative stress and inflammation can trigger senescence, also known as stress-induced premature senescence (SIPS). There is crosstalk between inflammation and senescence, forming a positive feedback loop. It is worth noting that premature cell senescence has become an important feature of AKI. Chen et al. (42) demonstrated that LXA4 inhibited NF-κB mediated inflammatory response and p53/p21 aging pathway in a PPAR-dependent manner, blocking the crosstalk between inflammation and premature senescence and exerting its renal protective effect.

Studies have shown that ferroptosis, a form of regulated cell death, is involved in various diseases, including tumors, neurodegenerative diseases, and ischemia-reperfusion injury. Ferroptosis is characterized by iron-dependent lipid peroxidation. GPX4 mediates the main cellular defense mechanism against it. Xiao et al. (14) first demonstrated that SAKI involved ferroptosis. After CLP, GPX4 expression in the kidney significantly decreased, while neutrophil gelatinase-associated lipocalin (NGAL) expression, a sensitive biomarker for AKI early diagnosis, significantly increased. However, pretreatment with ferroptosis inhibitor liproxstatin-1 or deferoxamine (DFO) reversed the increase in NGAL expression. It was further concluded that MCTR1 inhibited ferroptosis in CLP-induced AKI by upregulating nuclear factor erythroid 2-related factor 2 (Nrf2) expression.

Of note, sepsis survivors are also at significantly increased risk of progressing to chronic renal failure. AT-RvD1 has been shown to attenuate long-term renal dysfunction caused by a second insult after severe sepsis. This is based on finding that the levels of inflammatory factors such as IL-1β and TNF-α were reduced in septic-surviving mice treated with AT-RvD1 during a subsequent injury in a subacute AKI model. Additionally, pro-fibrotic markers such as transforming growth factor β (TGF-β), metalloproteinase (MMP)-3 and -9 were also significantly reduced after administration. It is well known that persistent inflammation, coupled with uncontrolled reparative processes, leads to excessive ECM deposition, resulting in interstitial fibrosis, which is a hallmark of progressive functional loss. AT-RvD1 confers renal protection against secondary injuries, including tubulointerstitial injuries and fibrosis induced by bovine serum albumin (BSA) after sepsis recovery (43). The protective mechanisms of SPMs against SAKI are detailed in **Table 2**.

## 4 Pharmacological effects of SPMs in sepsis-induced acute liver injury

The liver, with its roles in synthesis, metabolism, detoxification and immune defense, is susceptible to damage from various metabolites, microorganisms, and toxins. Sepsis-induced microcirculatory disorders can lead to intestinal mucosa ischemia, facilitating the translocation of endotoxins and bacteria via the portal vein, which in turn can impair liver function and trigger an inflammatory cascade. The pathogenesis of sepsis-induced acute liver injury likely involves alterations in hepatic blood flow and microcirculation, bacterial translocation, enhanced oxidative stress response, along with pro-inflammatory and immunosuppressive responses mediated by the liver.

D-galactosamine (D-GalN) has specific hepatotoxicity, and in LPS model of D-GalN-sensitized mice, the death of LPS/GalN mice was mainly caused by damage to the liver, but not to other organs, such as lungs. Studies indicated that SPMs, including LXA4 and RvD1, can mitigate apoptosis of liver parenchymal cell, decrease serum bacterial loads, and suppress inflammation by dose-dependently reducing levels of TNF-α, IL-6, IL-1β after LPS/D-GalN stimulation (34, 44, 45). Additionally, SPMs reduced the release of extracellular high mobility group box 1 (HMGB1), a critical late mediator in endotoxin shock. The anti-inflammatory action of SPMs involves dampening TLR4/NF-κB activation. Specifically, MaR1 upregulated the expression of Nrf2 and heme oxygenase-1 (HO-1) in a dose-dependent manner and inhibited phosphorylation of p38, ERK1/2, NF-κB p65 and IκBα, promoting an anti-inflammatory M2 macrophage phenotype in sepsis-induced acute liver injury mice. MaR1 exhibited anti-inflammatory and antioxidant properties (46).

Pyroptosis, confirmed in recent years as a form of programmed cell death, is biochemically characterized primarily by the formation of inflammasomes, the activation of caspases and gasdermin, and the release of a plethora of pro-inflammatory cytokines. In LPS/D-GalN-induced liver injury, MaR1 significantly reduced the expression of P30-GSDMD (gasdermin D) and inhibited pyroptosis induced by NLR family pyrin domain containing 3 (NLRP3) inflammasome (46). Furthermore, MaR1 mitigated D-GalN/LPS -induced liver injury in mice by attenuating ferroptosis, characterized by increased hepatic lipid peroxidation. Compared with the D-GalN/LPS group, MaR1 treatment increased serum GSH level and GSH/GSSG ratio in liver tissue while lowering serum iron levels. MaR1 also countered the rise in ROS, a key ferroptosis activator, and upregulated GPX4 expression. These effects were associated with the activation of Nrf2/HO-1/GPX4 pathways (47). NMR-based metabolomics studies have identified disruptions in metabolites related to energy metabolism and inflammation in sepsis models. MaR1 treatment alleviated metabolic disorders in CLP-induced septic mice, particularly affecting pyruvate, alanine, aspartate, glutamate, and pulmonary taurine metabolism (48). However, MaR1’s influence was observed in serum and lungs, not liver, possibly due to its temporal effect on sepsis. The hepatic metabolic response to MaR1 may require a more extended period for observable effects (48). The protective mechanisms of SPMs against sepsis-induced acute liver injury are detailed in **Table 3**.

## 5 Pharmacological effects of SPMs in sepsis-induced heart injury

Sepsis-induced heart injury is prevalent in severe sepsis, with up to 60% of septic shock patients experiencing cardiac dysfunction within the initial three days, which is predictive of mortality (49). The pathogenesis of septic cardiomyopathy remains elusive, and no targeted therapies are available (50, 51). SPMs may offer a potential therapeutic avenue against myocardial injury in sepsis by reducing inflammation, oxidative stress, and mitochondrial dysfunction (**Table 4**).

In a septic heart injury model induced by intraperitoneal injection of LPS, RvD1 and RvE1 preconditioning significantly improved cardiac function, as evidenced by enhanced left ventricular ejection fraction, and decreased serum myocardial injury markers such as lactate dehydrogenase (LDH) and creatine kinase myocardial bound (CK-MB). These treatments also reduced cardiac apoptosis and neutrophils/macrophages infiltration, modulated macrophage polarization, and suppressed pro-inflammatory cytokines secretion, via the MAPK and NF-κB pathways (52, 53). Additionally, RvD1 and RvE1 mitigated systemic inflammation by diminishing neutrophils and pro-inflammatory M1 macrophages infiltration in the spleen. Unlike the suppression of macrophages infiltration in the heart and spleen, RvE1 uniquely increased M2 macrophages in the peritoneum, correlating with enhanced bacterial clearance (54). This suggests that RvE1 differentially modulates macrophage responses to protect organs in the absence of bacteria and enhance bacterial clearance in infected sites.

Cardiac fibroblasts (CFs) are pivotal in the inflammatory response following cardiac injury, critical for tissue repair. RvD1 and RvE1 have demonstrated activity in CFs, inhibited LPS-induced inflammation by reducing the expression of ICAM-1 and VCAM-1 protein levels and lessening spleen mononuclear cells adhesion to CFs (55). This action promotes the resolution of inflammation, counteracting the sustained leukocyte adhesion that can lead to the release of pro-inflammatory mediators, ROS production, and subsequent cardiac fibrosis and remodeling. Interestingly, RvD1 inhibited LPS-induced elevations of IL-6, MCP-1, TNF-α, and IL-10, whereas RvE1 did not (55). The differential effect may be attributed to the distinct molecular structures of RvD1 and RvE1 and their potential interactions with corresponding receptors or signaling pathways. In addition, the effect of RvE1 on cytokine secretion appears to be tissue and/or cell type dependent and mediated by differences in the mechanisms associated with the receptors.

IL-17A is a pivotal cytokine in neutrophil recruitment, driving the production of chemotactic factors, including granulocyte colony-stimulating factor (G-CSF), chemokine (C-X-C motif) ligand 1 protein (CXCL1) and CXCL2 (56). It stimulates G-CSF to enhance neutrophils production and maturation, while CXCL1 and CXCL2 guide neutrophils to sites of infection or injury. In a pioneering study by Yang et al. (57), neutralization of IL-17A or depletion of γδT cells was shown to reduce neutrophil recruitment and ameliorate cardiac dysfunction in an LPS-induced cardiac injury. Additionally, MCTR1 was identified to improve cardiac function by mitigating neutrophil infiltration via the γδT/IL-17A signaling pathway (57). However, clinical studies on IL-17’s role in sepsis have yielded inconsistent results, with some indicating a weak positive correlation with disease severity in pediatric sepsis and others suggesting a beneficial role in survival (58, 59). Further in-depth study of the mechanisms is needed in future research.

MaR1 mitigated oxidative stress by reducing serum malondialdehyde (MDA) levels and elevating superoxide dismutase (SOD) and GSH levels in mice, as well as upregulating the expression of NRF-2 and HO-1 in cardiac tissues (60). The NRF2/HO-1 pathway is known for its antioxidant and anti-inflammatory properties, crucial in mitigating mitochondrial damage and regulating cellular processes. Mitochondrial dysfunction is increasingly recognized as the pathophysiological core of sepsis-induced cardiac dysfunction. peroxisome proliferator-activated receptor (PPAR)-γ coactivator-1α (PGC-1α), a key regulator in mitochondrial biosynthesis, promotes the expression of NRF1, NRF2, and TFAM, enhancing mitochondrial function. SIRT1 has been implicated in modulating PGC-1α-mediated mitochondrial biogenesis. LPS-induced mitochondrial dysfunction was characterized by decreased membrane potential and reduced activity of mitochondrial complexes and ATP synthesis. Post-treatment of MCTR1 dose-dependently increased SIRT1 expression, leading to enhanced PGC-1α and its target factors, thereby improving mitochondrial biogenesis and function, and protecting against sepsis-induced cardiac dysfunction (61).

## 6 Pharmacological effects of SPMs in sepsis-induced brain injury

Sepsis-induced brain injury, also known as sepsis-associated encephalopathy (SAE), is characterized by diffuse brain dysfunction secondary to sepsis, manifesting as neurological symptoms such as dementia, delirium, and even long-term cognitive impairment. It typically lacks overt central nervous system infection or structural lesions. The etiology of SAE remains elusive, with uncertainty as to whether it arises from a cascading response to multi-organ dysfunction or is precipitated by inflammation caused by sepsis early (62). The pathophysiology of SAE is intricate, potentially involving blood-brain barrier disruption, neuroinflammation, mitochondrial dysfunction, and neurotransmitter (63).

Microglia, the brain’s innate immune cells, are implicated in the pathogenesis of SAE through their inflammatory response. Upon activation by inflammatory stimuli, these cells can protect neurons but may also induce neuronal death and tissue damage through excessive production of cytotoxic factors such as superoxide, nitric oxide, TNF-α, and IL-1β. In the LPS-stimulated BV-2 cells study, AT-LXA4 inhibited the production of NO, IL-1β and TNF-α in a concentration-dependent manner, at least partially through NF-κB, ERK, p38 MAPK and activator protein-1 (AP-1) pathways (64). Similarly, RvD1 was found to attenuate microglia activation in the hippocampus of SAE mice, thereby reducing inflammatory mediators such as TNF-α, IL-6, and IL-1β and enhancing cognitive functions. These effects were likely mediated by the suppression of NF-κB, MAPK, and STAT pathways (65). Interestingly, in an LPS-stimulated BV2 cell model, Rey et al. (66) demonstrated that RvD1 and RvE1 inhibited LPS-induced TNF-α, IL-6 and IL-1β gene expression and regulated microglia phenotype with distinct underlying mechanisms. RvE1 downregulated the NF-κB signaling pathway, while RvD1 regulated the expression of miRNAs. Following RvD1 treatment, the expression of miR-155, miR-21, and miR-146 was upregulated, while that of miR-219 was downregulated. miR-155 targeted proteins are involved in NF-κB activation, potentially controlling tissue damage associated with inflammation (67). The miR-21, a key player in inflammatory response, was crucial for the resolution of inflammation and the negative regulation of pro-inflammatory responses, particularly in macrophages (68). The miR-146, a microRNA with negative regulatory function, is implicated in the regulation of immune response (69). The mechanisms by which SPMs exert their influence on neuroinflammation in SAE remain to be fully elucidated and warrant further investigation. The protective mechanisms of SPMs against sepsis-induced brain injury are detailed in **Table 5**.

## 7 SPMs in sepsis

**Figure 1:**
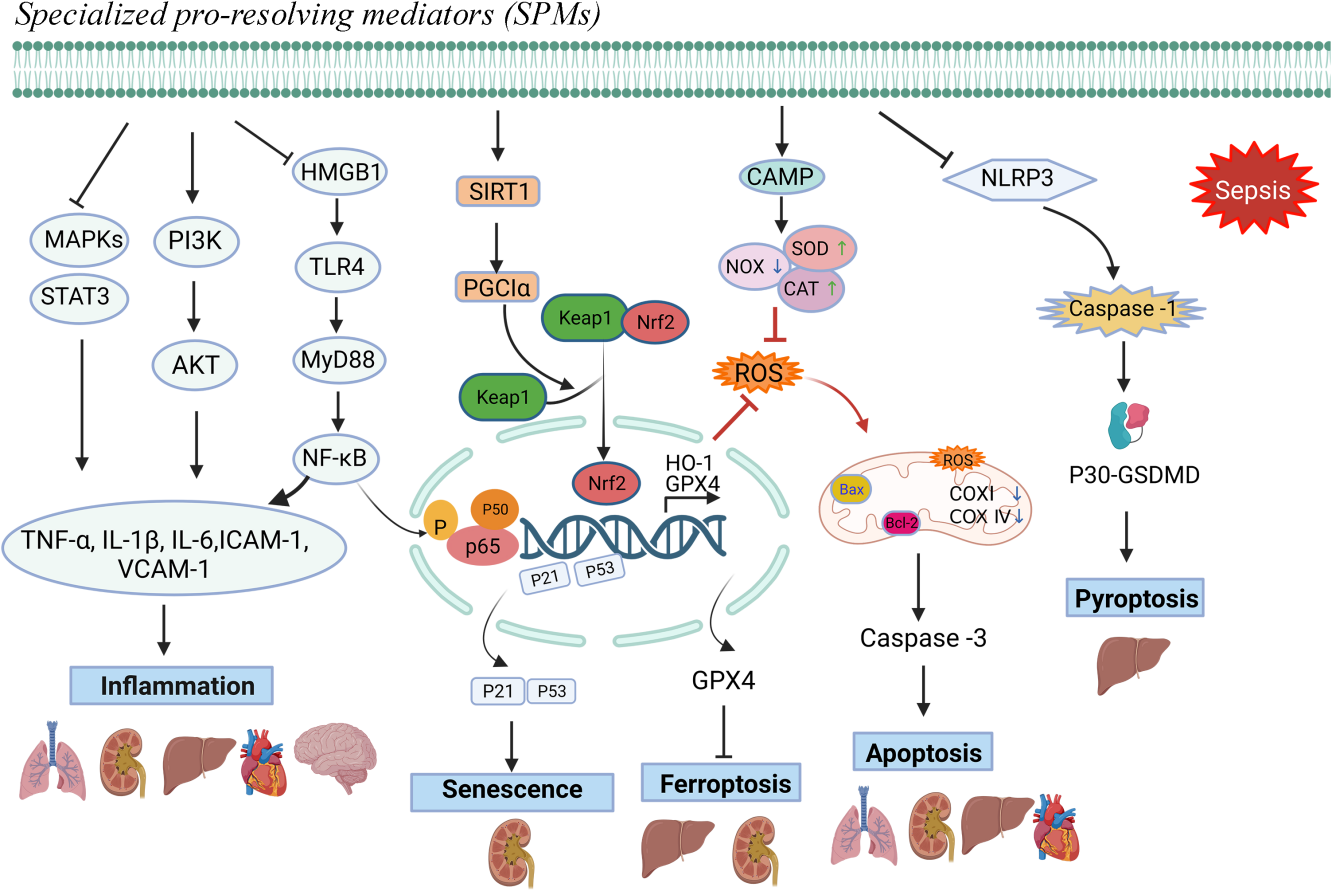
Protective mechanisms of SPMs against sepsis-induced organ dysfunction.Note: Specialized pro-resolving mediators (SPMs) can modulate inflammatory mediators, mitigate oxidative stress responses, and counteract cellular damage mechanisms, including apoptosis, ferroptosis, pyroptosis, and senescence, thereby facilitating the resolution of inflammation and preventing sepsis-induced organ damage.

SPMs appear to possess similar anti-inflammatory and pro-resolving effects in various sepsis-induced organ injuries, as evidenced by the current literature. Firstly, SPMs inhibit the production of pro-inflammatory cytokines and chemokines, which are key mediators in the inflammatory process, thereby mitigating tissue damage and organ dysfunction; Secondly, SPMs can accelerate the apoptosis and clearance of inflammatory cells, and also stimulate the repair and regeneration of damaged tissues. Thirdly, while reducing inflammation, SPMs also help to bolster the host’s defense mechanisms against pathogens by maintaining the function of immune cells and promoting their ability to eliminate invading microorganisms. Overall, SPMs exhibit anti-inflammatory activity, promote the resolution of inflammation, and modulate immune responses in septic organ damage, which helps to attenuate inflammatory damage and promote tissue repair. The relevant mechanisms involved are shown in **Figure 1**. Specifically, immunosuppression is frequently linked to mortality from sepsis. Unlike some anti-inflammatory drugs that carry a risk of immunosuppression, SPMs may be more appropriate for treating patients with sepsis because they suppress inflammation while enhancing host defense. Some studies suggested that SPMs may play a beneficial role in late-stage sepsis. RvD2 (administered late in an infection) improved bacterial clearance and increased survival in mice in a 2 - hit model of sepsis with secondary lung infection (70, 71). RvD2 was shown to directly increase macrophage phagocytosis and increase the number of M-MDSC in the spleens of CLP mice, which are the primary mechanisms for bacterial clearance in late-stage sepsis (30, 71). The ability to eradicate the source of infection while maintaining immunological balance provides an attractive therapeutic modality for sepsis. To clarify the precise role of SPMs during various stages of sepsis, more research would be worthwhile.

There is some controversy about the specificity of detection methods for quantitative analysis of SPMs in biological samples. For example, Chen et al. (54) first reported that RvE1 levels were significantly reduced in SAHI mice (by~93-fold). However, this applied method has recently been seriously questioned (72). The LC-MS method in this paper (54) does not conform to internationally applied rules which use the S/N ratio for the LLOQ in LC-MS. Indeed, in another case, reanalysis of data obtained with this method by application of the generally accepted S/N ratio method revealed that the samples do not contain SPMs (73). The SPM profile in sepsis patients has also been studied in some human clinical trials. Dalli et al. (74) suggested that SPMs (RvE1, RvD5, and 17R-PD1) were detected at increased levels in the peripheral blood of those who succumbed to sepsis. The study also suggested that PDX at day 3 was more predictive of ARDS development than APACHE II scores (74). However, the same issue persists; the paper employed a nonconforming LC/MS method, and the data have to be interpreted with care. Plasma specimens from 18 cases of early septic shock (<12 h) revealed a trend towards higher SPMs in septic patients. Nevertheless, the authors concluded that this experiment did not reveal a significant correlation between SPMs levels and severity of sepsis, or days of survival (75). On the contrary, a study found that the SPM concentration in sepsis patients was lower than that in the healthy control group, but the measurement method used, ELISA, was clearly inappropriate (76). There is insufficient evidence to establish a correlation between endogenous SPM levels and clinical outcomes in sepsis. Clearly, the temporal distribution of SPMs levels in sepsis patients and their biological roles are complex. Furthermore, it is undeniable that consistently reported levels of SPMs are typically very low (77), and it is clearly challenging to reliably quantify SPMs levels in biological samples. The wide variation in SPM concentration within and between studies may be related to differences in sample storage, sample handling and analysis, but has not yet been fully explained. Accurate measurement techniques are critical to understanding the role of SPMs in sepsis and its potential clinical implications. Further research is needed to use highly reliable analytical methodology to determine the clinical relevance of SPMs to sepsis, as the current evidence is inconclusive.

## 8 Discussion

As we mentioned previously, concerns have been raised about some of the key data emerging from studies of SPMs. The biosynthetic pathway of most SPMs involves 5-LOX (ALOX5) as well as 12/15-LOX activity. Biochemical studies with purified lipoxygenases have shown that their ability to form trihydroxylated SPMs is extremely low, whereas the ability to form dihydroxylated SPMs is much lower than the rate of classical inflammatory response products (e.g. leukotrienes). Furthermore, to the best of our knowledge, knockout of ALOX12 or ALOX15 in mice which prevents SPMs formation does not lead to significant defects in inflammation resolution. Elimination or blockade of 12/15-lipoxygenase reduced neutrophil recruitment and improves survival in a mouse model of ALI (78). A loss-of-function variant in ALOX15 protected against nasal polyps and chronic rhinosinusitis in humans (79). However, anti-inflammatory effects of this enzyme have also been demonstrated in several reports. Corneal wound healing was delayed in eosinophile-specific 12/15-LOX knockout mice compared to wild-type mice (80). In the study by Pernet et al. (81), ALOX15-/- mice exhibited a heightened inflammatory response in alveolar macrophages and more severe lung inflammation and injury following LPS stimulation compared with wild-type mice. Notably, the authors attributed this effect to the formation of 12-HETE, not SPMs. These observation underscore the complexity of lipid mediators in the inflammatory process and highlights the need for a nuanced understanding of their roles in inflammation. The absence of its putative biosynthetic enzymes fails to provide evidence consistent with a role for SPM in resolving inflammation. At the same time, there is also no clear evidence that any of the described biological effects caused by lipoxygenase knockout are due to disruption in SPMs formation. Evidence for endogenous and pharmacological properties of SPMs remains controversial and incomplete.

As we have summarized from the literature, SPMs appear to have tremendous potential in alleviating organ damage in sepsis. The wide range of drug dosages used across studies, as well as the different routes of administration, are also noteworthy. It is indeed a valid concern that the concentrations used, such as 1 ng/mouse, may seem insufficient to induce a biological effect. Given the findings that suggest certain SPM receptors may not be functional, it is crucial to critically assess whether these low concentrations can indeed exert a significant effect on inflammation. Moreover, considering the nature of oxylipins and the potential for rapid metabolism, the persistence and bioavailability of SPMs at these low doses become even more questionable. It is crucial to consider the possibility that the observed effects may be due to indirect actions, alternative receptors, or other yet unidentified mechanisms. Future studies should aim to clarify the dose-response relationship of SPMs and validate their functional receptors to ensure that the concentrations used are biologically relevant and can effectively contribute to inflammation resolution. Furthermore, it is necessary to conduct further experiments, including pharmacokinetic analysis and tissue distribution studies. These data will help us to more accurately estimate the SPM concentration required to produce therapeutic effects *in vivo* and may guide us in adjusting the dosage to optimize therapeutic efficacy. Addressing these concerns will be crucial in advancing the field of resolution pharmacology and translating these findings into effective clinical applications.

It remains to be identified that the concentration of SPMs formed in the body correlates with the resolution of inflammation. Studies detecting these molecules within the human body typically report only low concentrations, potentially insufficient to significantly advance the resolution of inflammation, especially when juxtaposed with the notably higher levels of pro-inflammatory mediators (9). Evidence for the formation of biologically active concentrations of SPMs in humans to promote inflammation resolution remains to be provided.

However, the current consensus, at least with respect to SPMs, is that high concentrations of SPMs chemical entities do have a role in modulating the inflammatory response, and even though natural concentrations of SPMs do not eliminate inflammation, this has not deterred drug developers from synthesizing SPMs molecules and exploring their clinical applications (82). At least three biotech companies are currently planning clinical trials with SPMs synthetic molecules meant to restore or boost the body’s natural ability to end inflammation, which can wreak havoc if prolonged. There is already a phase I clinical trial of a lipoxin-based mouthwash, which has been shown to be safe for patients with periodontal disease and has also shown some efficacy, while Thetis Pharmaceuticals is planning to conduct clinical trials of a synthetic resolvin for the treatment of inflammatory bowel disease and cancer (82).

The results cited in our review were obtained mainly on the basis of preclinical modelling studies. The models of sepsis used in most studies are the LPS model and the CLP model. The LPS model reflects a systemic inflammatory response induced by a single pathogen component, primarily through LPS-induced activation of immune cells leading to a massive release of inflammatory factors. In contrast, the CLP model involves multiple bacterial infections and a more complex host response, including local and systemic inflammation, and subsequent immune modulation. The CLP model is better able to simulate the multi-organ damage and dysfunction of sepsis, including the lungs, kidneys, liver and heart. These differences are equally important for understanding the potential role of SPMs in sepsis treatment. Although these models are valuable in exploring mechanisms, they are poor predictors of potential clinical applications. SPMs have been used as pharmacological tools for decades, but their clinical development has been relatively slow, which may reflect the potential limitations of these compounds in clinical applications. More basic and clinical studies are needed in the future to answer and enrich the mode of action of SPMs in sepsis.

## 9 Conclusion

Sepsis is often characterized by an uncontrolled inflammatory response and immunosuppression. The resolution of inflammation is now recognized as an active process. SPMs exhibit anti-inflammatory, pro-resolving and immunomodulatory properties. A large body of evidence suggests that it may be a potential treatment for sepsis-induced organ dysfunction. However, evidence for endogenous and pharmacological properties of SPMs remains controversial and incomplete. Therefore further studies need to be conducted to clarify the role of SPMs in different stages of sepsis. As the field progresses, the translation of SPMs into clinical applications may provide new therapeutic strategies for sepsis, focusing not only on controlling inflammation but also on restoring immune homeostasis and organ function.
